# Clinical Risk Factors of Death From Pneumonia in Children with Severe Acute Malnutrition in an Urban Critical Care Ward of Bangladesh

**DOI:** 10.1371/journal.pone.0073728

**Published:** 2013-09-09

**Authors:** Mohammod Jobayer Chisti, Mohammed Abdus Salam, Hasan Ashraf, Abu S. G. Faruque, Pradip Kumar Bardhan, Md Iqbal Hossain, Abu S. M. S. B. Shahid, K. M. Shahunja, Sumon Kumar Das, Gazi Imran, Tahmeed Ahmed

**Affiliations:** 1 Centre for Nutrition & Food Security (CNFS), International Centre for Diarhoeal Disease Research, Bangladesh (icddr,b), Dhaka, Bangladesh; 2 Dhaka Hospital, icddr,b, Dhaka, Bangladesh; 3 Research Administration, icddr,b, Dhaka, Bangladesh; 4 Dhaka Shishu (Children) Hospital, Dhaka, Bangladesh; University of Alabama at Birmingham, United States of America

## Abstract

**Background:**

Risks of death are high when children with pneumonia also have severe acute malnutrition (SAM) as a co-morbidity. However, there is limited published information on risk factors of death from pneumonia in SAM children. We evaluated clinically identifiable factors associated with death in under-five children who were hospitalized for the management of pneumonia and SAM.

**Methods:**

For this unmatched case-control design, SAM children of either sex, aged 0–59 months, admitted to the Dhaka Hospital of the International Centre for Diarrhoeal Disease Research, Bangladesh (icddr,b) during April 2011 to July 2012 with radiological pneumonia were studied. The SAM children with pneumonia who had fatal outcome constituted the cases (n = 35), and randomly selected SAM children with pneumonia who survived constituted controls (n = 105).

**Results:**

The median (inter-quartile range) age (months) was comparable among the cases and the controls [8.0 (4.9, 11.0) vs. 9.7 (5.0, 18.0); p = 0.210)]. In logistic regression analysis, after adjusting for potential confounders, such as vomiting, abnormal mental status, and systolic hypotension (<70 mm of Hg) in absence of dehydration, fatal cases of severely malnourished under-five children with pneumonia were more often hypoxemic (OR = 23.15, 95% CI = 4.38–122.42), had clinical dehydration (some/severe) (OR = 9.48, 95% CI = 2.42–37.19), abdominal distension at admission (OR = 4.41, 95% CI = 1.12–16.52), and received blood transfusion (OR = 5.50, 95% CI = 1.21–24.99) for the management of crystalloid resistant systolic hypotension.

**Conclusion and Significance:**

We identified hypoxemia, clinical dehydration, and abdominal distension as the independent predictors of death in SAM children with pneumonia. SAM children with pneumonia who required blood transfusion for the management of crystalloid resistant systolic hypotension were also at risk for death. Thus, early identification and prompt management of these simple clinically recognizable predictors of death and discourage the use of blood transfusion for the management of crystalloid resistant systolic hypotension may help reduce deaths in such population.

## Introduction

Over the last two decades, pneumonia remained the leading cause of global under-five childhood deaths [1], [2], representing an estimated 1.4 million out of the total 7.6 million deaths in this population in 2010 [3]. The risk of death is high when children with pneumonia have the co-morbidity of severe acute malnutrition (SAM) [4], [5] and has been reported to be 15 times higher compared to deaths in children who did not have SAM [6]. Children with severe wasting or severe under-nutrition, or nutritional edema were considered as SAM. Most of these pneumonia and diarrhea related deaths in SAM children occur in the critical care wards of developing countries [4]. However, clinical features of pneumonia in children with SAM often remain subtle [6], [7]. Health workers, particularly in resource constrained settings may be less confident in identifying clinical features for the diagnosis of pneumonia in SAM children and as a result they might offer only oral antibiotics following recent WHO recommendations if the SAM children do not have any complications [8]. The bacterial pathogens causing pneumonia in SAM children are often different than those in better-nourished children [6], [9], [10]. Therefore, the subtle clinical signs and different etiology of pneumonia in SAM children may necessitate first dose of parenteral antibiotics before their referral to tertiary hospitals with the objectives to reduce morbidity and death. However, this management approach might not be feasible at every health care facility in resource limited settings due to lack of funds. From this perspective, identification of simple clinical cues for fatal outcome in SAM children with pneumonia may prove very useful to health professionals, particularly health workers in making referral decisions. However, there is lack of information on factors predictive of death in such children. With these contexts, we planned our study to identify simple clinically recognizable predictors of death in hospitalized, under-five SAM children with pneumonia.

## Materials and Methods

### Ethics Statement

The study (protocol number: PR-10067) was approved by the Research Review Committee (RRC) and the Ethical Review Committee (ERC) of International Centre for Diarhoeal Disease Research, Bangladesh (icddr,b). A written informed consent was obtained from parents/caregivers of all participating children. Children whose parents/caregivers did not provide consent were not included in the study.

### Study Design

This unmatched case control study was conducted at the Dhaka Hospital of icddr,b. SAM children of either sex, aged 0–59 months, admitted to the Intensive Care Unit (ICU) of the Dhaka Hospital of the International Centre for Diarrhoeal Disease Research, Bangladesh (icddr,b) during April 2011 to July 2012 with radiological pneumonia were eligible. SAM children with pneumonia who had fatal outcome constituted the cases, and SAM children with pneumonia who survived constituted controls. Controls were randomly selected by computer randomization using SPSS (version 17.0; SPSS Inc, Chicago) from a personal computerized data source of this study. This database identified 370 controls, and 1∶3 unmatched case-control ratios were used to increase the statistical power of our analyses. Pneumonia was defined radiologically as the presence of end-point consolidation or other (non-end-point) infiltrate in lungs according to the WHO radiological classification of pneumonia [11] and the finding was confirmed independently by a qualified radiologist (FBM) and a pediatric respiratory physician (MJC). When there was any disagreement on radiologic evidence of pneumonia we did not include them in our study. Children with severe wasting [weight for height z score (WHZ)<−3 of the median of the WHO anthropometry] or severe under-nutrition [weight for age z score (WAZ)<−4 of the median of the WHO anthropometry], or nutritional edema were considered as SAM.

### Setting

The Dhaka Hospital of icddr,b provides care and treatment to around 140,000 patients of all ages and either sex with diarrhea, with or without associated complications or health problems. Diarrhea and/or acute respiratory infection (ARI) are the entry points for admission to the Dhaka Hospital of icddr,b. Children with complications of diarrhea, or those with respiratory distress, cyanosis, apnea, hypothermia, sepsis, shock, impaired consciousness, convulsion, severe/very severe pneumonia with hypoxemia or respiratory failure are admitted to the ICU of the hospital. The vast majorities of the patients visiting the hospital have poor socio-economic background and most live in urban and peri-urban Dhaka.

### Patient Management

Patients admitted to the ICU receive standardized care and treatment, following hospital guidelines that include antibiotic therapy, supportive care such as intravenous fluids and oxygen, frequent monitoring, and nutritional support (breast milk, formula, solid and semisolid diets, micronutrients, and zinc). Mechanical ventilation is used for management of children admitted to ICU with respiratory failure. All children in the study were assessed by the attending ICU physicians, who recorded medical history, performed clinical examinations, and determined management plan. Arterial oxygen saturation (SpO_2_) was measured using a portable pulse oximeter (OxiMax N-600, Nellcor, Boulder, CO) and blood glucose was estimated using a bedside Gluco-check machine (STADA, Bad Vilbel, Germany).

Children with hypoxemia received O_2_ supplementation through nasal prongs (2 L/min) or mask (5 L/min). Antibiotics were prescribed for children with pneumonia, sepsis, severe cholera, dysentery, severe malnutrition, and other bacterial infections. Dehydration was corrected using ORS solution, orally or through NG tube, or appropriate intravenous fluid when dehydration was severe or when children had severe respiratory distress. Pneumonia was managed according to the WHO algorithm (19) and management of severe protein-energy malnutrition (PEM) was done following the hospital guidelines [12], [13].

### Measurements

Case report forms (CRF) were developed, pretested, and finalized for acquisition of study relevant data. Characteristics analyzed included demographic information (age, gender, residence, socio-economic status, working mother, lack of vaccinations, non-breast-feeding), clinical signs {AWD, vomiting, dehydration (defined by “Dhaka methods” of assessment of dehydration that is almost similar to WHO method and approved by WHO [14]), nutritional edema, WHZ and WAZ, abnormal mental status (irritable/lethargy/convulsion), abdominal distension, hypoxemia [arterial oxygen saturation (SPO_2_) <90% in air [8]], systolic hypotension (<70 mm of Hg), refractory/crystalloid resistant systolic hypotension [unresponsive to crystalloid, i.e. unresponsive to 20 ml per kg per hour physiological saline (sodium: 154 mMol/L and chloride: 154 mMol/L) or cholera saline (sodium: 133 mMol/L, potassium: 13 mMol/L, chloride: 98 mMol/L, acetate: 48 mMol/L) [5] (maximum 40 ml over 2 hours)], heart failure (defined as tachypnea, tachycardia, enlarged tender liver, gallop, basal rales, non-pitting edema)}, blood transfusion, hypoglycemia and hematocrit (Hct%). All these variables except blood transfusion and heart failure were the admission characteristics at ICU of the Dhaka Hospital of icddr,b.

### Analysis

All data were entered into SPSS for Windows (version 15.0; SPSS Inc, Chicago) and Epi-Info (version 6.0, USD, Stone Mountain, GA). Differences in proportion were compared by the Chi-square test. Student’s t-test was used to compare the means of normally distributed data and Mann-Whitney test was used for comparison of data that were not normally distributed. A probability of less than 0.05 was considered statistically significant. Strength of association was determined by calculating odds ratio (OR) and their 95% confidence intervals (CIs). In identifying risks for death in children with SAM and pneumonia, variables were initially analyzed in a uni-variate model, and then predictors independently associated with deaths were identified using logistic regression after controlling for the co-variates.

## Results

There were 35 cases and 105 controls. Cases more often had vomiting, abnormal mental status, and systolic hypotension (<70 mm of Hg) in absence of dehydration or after correction of existing dehydration compared to the controls (Table 1). Cases more often presented with lower Hct% on admission compared to controls (27.7±6.1 vs. 31.5±6.1; p<0.001), but admission Hct% of the children who received blood transfusion was comparable among the cases and the controls (26.3±6.6 vs. 20.8±8.4; p = 0.176). In logistic regression analysis, after adjusting for potential confounders, such as vomiting, abnormal mental status, and systolic hypotension in the absence of or after correction of existing dehydration, under-five SAM children with pneumonia more often had hypoxemia, clinical dehydration (some/severe) and abdominal distension at admission, and received blood transfusion (Table 2). The distribution of age, WAZ, WHZ, sex, residence, socio-economic status, working mother, lack of vaccinations, non-breast-fed, AWD, nutritional edema, hypoglycemia diagnosed at bedside, and heart failure were equally distributed among the cases and the controls (Table 1).

**Table 1:** Clinical characteristics of under-five children having pneumonia and severe acute malnutrition with (cases) and without fatal outcome (controls).

Characteristic	Cases (n = 35)	Controls (n = 105)	OR	95% CI	p
Male sex	23 (66)	62 (59)	1.33	0.56–3.19	0.617
Age in months (median, IQR)	8.0 (4.9, 11.0)	9.7 (5.0, 18.0)	–	–	0.210
Resides outside Dhaka District	11 (31)	18 (17)	2.22	0.84–5.79	0.118
Poor socio-economic condition	30 (86)	86 (82)	1.33	0.42–4.47	0.796
Working mother	10 (29)	42 (40)	0.60	0.24–1.48	0.313
BCG vaccination not received	2 (6)	12 (11)	0.47	0.07–2.40	0.517
DPT/oral polio/Hib/Hepatitis vaccination not received	5 (14)	27 (26)	0.48	0.15–1.48	0.245
Measles vaccine not received	8 (23)	30 (29)	0.74	0.27–1.96	0.661
Non-breastfed (up to 6 months of age)	5 (14)	17 (16)	0.86	0.25–2.78	1.00
Acute Watery Diarrhea	31 (89)	82 (78)	2.17	0.64–8.10	0.266
Children with vomiting	12 (34)	15 (14)	3.13	1.18–8.32	0.019
Clinical dehydration (some/severe)	16 (46)	9 (9)	8.98	3.16–26.16	<0.001
Nutritional edema	5 (14)	5 (5)	3.33	0.77–14.50	0.121
WHZ (mean ± SD)	−3.75±1.83	−3.73±1.37	−0.02*	−1.28–0.06	0.943
WAZ (mean ± SD)	−5.58±1.69	−4.98±1.74	−0.61*	−0.60–0.56	0.073
Abnormal mental status	19 (54)	12 (11)	9.20	3.45–25.06	<0.001
Abdominal distension	10 (29)	10 (10)	3.80	1.29–11.30	0.012
Presence of hypoxemia	14 (40)	3 (3)	22.67	5.39–110.01	<0.001
Systolic hypotension (<70 mm of Hg) aftercorrection of dehydration or in absenceof dehydration	6 (18)	4 (4)	5.61	1.28–25.90	0.012
Hypoglycemia (random blood sugar <3.0 mmol/L)on admission	2 (6)	1 (1)	6.30	0.43–181.82	0.154
Blood transfusion done for management of refractory systolic hypotension (<70 mm of Hg even after40 ml/kg bolus fluid)	11 (31)	5 (5)	9.17	2.61–33.92	<0.001
Heart failure	1 (3)	3 (3)	1.0	(0.18–5.60)	1.00

Figures represent n (%), unless specified. OR: odds ratio. CI: confidence interval. IQR: inter-quartile range.BCG: Bacillus Calmette-Guerin. DPT: Diphtheria, Pertusis and Tetanus. Hib: Hemophilus Influenza type B. SD: standard deviation. WHZ: weight for height z score; WAZ: weight for age z score; SpO_2:_ transcutaneously measured blood oxygen concentration;

* mean difference.

**Table 2:** Results of logistic regression to explore the independent risks of fatal outcome in under-five children with pneumonia and severe acute malnutrition.

Characteristics	OR	95% CI	p
Abnormal mental status	1.84	0.45–7.60	0.400
Children with vomiting	1.09	0.25–4.74	0.912
Clinical dehydration (some/severe)	9.48	2.42–37.19	0.001
Abdominal distension	4.41	1.12–16.52	0.028
Presence of hypoxemia	23.15	4.38–122.42	<0.001
Systolic hypotension (<70 mm of Hg) after correction of dehydration or in absence of dehydration	1.50	0.17–13.59	0.721
Blood transfusion used for the management of refractory systolic hypotension (<70 mm of Hg)	5.50	1.21–24.99	0.027

## Discussion

We observed that blood transfusion used for the management of refractory systolic hypotension revealed as the independent predictor for death in under-five SAM children with pneumonia – a very important information for clinicians in critical care wards of developing countries. WHO recommends blood transfusion in severely malnourished children who do not recover from septic shock even after infusion of consecutive two boluses of isotonic fluid [8]. The protocolized management of such children in our hospital followed this recommendation [13]. Systolic hypotension, in addition to features of sepsis (defined by our local guideline) [15], [16], are used as the marker of septic shock in SAM children, especially in resource limited settings. Children with systolic hypotension and unresponsive to crystalloid received blood transfusion but did not receive diuretics and had frequent fatal outcome. We do not have any ready explanation for this finding. All of our study children received blood transfusion due to septic shock refractive to fluid therapy, which might be due to septic myocardial dysfunction characterized by decrease in ejection fraction with dilatation of ventricles [17]–[19]. Death in this special population is often very high even with adequate treatment not only in developing countries [16] but also in developed countries [20]. However, the impact of blood transfusion on deterioration in heart function in SAM children is unclear to us. Recent data suggest that reduction of alveolar epithelial sodium and chloride transport in pneumonic SAM children impedes clearance of fluid from the alveolar exudates [21], [22]. This may contribute to development of interstitial edema/heart failure in our study children who received blood transfusion in addition to receiving crystalloid fluids. However, clinical evidence of fluid overload/heart failure was not different among the cases and controls. Thus, pulmonary edema, a common etiology for death in pneumonic children with SAM [23], might not be responsible for the detrimental effect of blood transfusion in our study population. Although an earlier study conducted in Mulago hospital, Uganda experienced significant higher deaths after blood transfusion related to pulmonary edema in SAM children compared to those who did not receive blood transfusion, most of the indications of blood transfusions in that study were other than septic shock and often the use of blood transfusion was not judicious [24]. We did not evaluate the cardiac function of these children to exclude fluid overload as a consequence of blood transfusion. A recent study has reported cardiovascular collapse rather than fluid overload to contribute to excess death from rapid fluid resuscitation in well nourished children with septic shock [25]; however, cardiac function in SAM children with septic shock has not been explored yet, which needs to be addressed in carefully conducted patho-physiologic studies in future.

Role of blood transfusion to increase inflammation such as with transfusion-related acute lung injury (TRALI) in children is rare [26]. Although extensive screening of donor’s blood and cross matching with donor blood were performed before transfusion, the remote chances of association of death with TRALI in our study population could not be ruled out.

Although, admission Hct% was significantly lower in fatal cases than the survivors, Hct% of the children who received blood transfusion was comparable among the fatal cases and the survivors which indicate that Hct% might not have any impact on case-fatality among the children who received blood transfusion.

Systolic hypotension after adequate rehydration along with replacement of ongoing fluid losses is likely to be secondary to impaired vaso-regulation and cardiac function as a result of sepsis and is often associated with death [27], [28], which is similar to our earlier observations [16], [29]. This event was significantly associated with deaths in univariate analysis (Chi-square test) but after adjusting for the potential confounders in logistic regression, it failed to remain as an independent predictor for death in under-five SAM children with pneumonia, but blood transfusion remained as one of the independent predictors for death in such children indicating strong association of blood transfusion with deaths. Although it seems like blood transfusion is a proxy for late treatment of systolic hypotension, all the children with systolic hypotension were quickly recognized, promptly managed with crystalloid (20 ml per kg per hour physiological saline or cholera saline) and blood transfusion was only given in crystalloid resistant systolic hypotension. The finding underscores the importance and urgency of conducting randomized clinical trial to evaluate the true effect of blood transfusion in such children.

Our observation of strong association between admission hypoxemia, clinical dehydration (some/severe), and abdominal distension in pneumonic under-five SAM and fatal outcome are understandable. In pneumonic children, hypoxemia may occur as a consequence of impairment of alveolar-arterial oxygen diffusion and concomitant increase in the partial pressure of carbon-dioxide (CO_2_) due to abnormally lower alveolar ventilation [30], [31]. This phenomenon in SAM children with pneumonia represents very severe illness often with fatal outcome. The association of hypoxemia and death has been reported by a number of earlier studies without describing nutritional status of the children [32], [33]. Abdominal distension is one of the common consequences of severe form of sepsis [34] due to compromised splanchnic circulation, often leading to paralytic ileus and death [35], [36]. Clinical dehydration is also associated with poor peripheral circulation [37], which might aggravate myocardial dysfunction in the septic SAM children with fatal outcome.

The observation of indifference in distribution of baseline characteristics on admission such as age, WAZ, WHZ, sex, residence, socio-economic status, working mother, lack of vaccinations, non-breast-fed, AWD, nutritional edema, and bedside hypoglycemia potentially eliminates the chances of bias in selection of controls and thus validate the study results.

In conclusion, the results of our data suggest that under-five SAM children with pneumonia who had hypoxemia, clinical dehydration, abdominal distension at admission, and those who require blood transfusion for the management of crystalloid resistant systolic hypotension during the course of hospitalized treatment are at higher risk of death. Identification of these simple, clinically recognizable features in such children may alert health professionals, especially health workers to administer the first dose of parenteral broad spectrum antibiotics before their referral to the critical care medicine words. The clinicians in the critical care ward should be discouraged in using blood transfusion for the management of crystalloid resistant systolic hypotension in an effort to reduce morbidity and deaths in such population, especially in resource limited settings. Carefully conducted, randomized clinical trial with adequate sample size is required to evaluate the impact and the role of blood transfusion from patho-physiologic point of view in the management of septic shock in SAM children who also have pneumonia.
